# Photodynamic inactivation increases cell death rate on persistent *Staphylococcus aureus*

**DOI:** 10.1111/php.70036

**Published:** 2025-09-15

**Authors:** Maria Vitória Silva Pereira, Bruna Carolina Corrêa, Vanderlei Salvador Bagnato, Kate Cristina Blanco

**Affiliations:** ^1^ São Carlos Institute of Physics University of Sao Paulo São Paulo Brazil; ^2^ Biomedical Engineering Texas A&M University College Station Texas USA

**Keywords:** curcumin, oxacilin, Persistence, Photodynamic Inactivation, *Staphylococcus aureus*

## Abstract

Bacterial persistence is characterized by a subpopulation of metabolically dormant cells that exhibit transient tolerance to antibiotics, contributing to chronic and recurrent infections, particularly in *Staphylococcus aureus*, a pathogen responsible for severe infections. This phenomenon is evidenced by a biphasic killing curve, where an initial rapid decline is followed by a slowed death phase. Photodynamic inactivation (PDI) represents a promising strategy for microbial eradication through the generation of reactive oxygen species (ROS). This study investigated persistence formation in two *S. aureus* strains and evaluated the effects of PDI using curcumin. Time‐kill assays with oxacillin revealed biphasic killing curves, indicative of persistence. Heritability testing confirmed that persistence was not passed on to progeny, supporting its phenotypic nature. PDI was performed using curcumin and blue light (450 nm), resulting in a dose‐dependent reduction in bacterial viability. However, populations that survived PDI exhibited tolerance‐like behavior, with unchanged MIC values, suggesting that ROS generated during PDI may induce a transient dormant state. Notably, post‐PDI time‐kill assays conducted after metabolic recovery showed a higher rate of bacterial death, indicating enhanced antibiotic susceptibility. In contrast, methicillin‐resistant strains (MRSA) showed limited persistence induction, likely due to enhanced oxidative stress defenses. These are important to the understanding of bacterial physiological states when designing therapeutic strategies. The timing of antibiotic administration relative to PDI treatment plays a crucial role in treatment efficacy, which may be either enhanced or compromised depending on bacterial adaptation and recovery dynamics.

## Graphical abstract

Persistent bacteria have the ability to survive extended periods of antibiotic stress by entering a metabolically dormant state. This study investigated persistence formation in two *S. aureus* strains and evaluated the effects of PDI using curcumin. Post‐PDI time‐kill assays conducted after metabolic recovery showed a higher rate of bacterial death, indicating that the timing of antibiotic administration relative to PDI treatment plays a crucial role in treatment efficacy, which may be either enhanced or compromised depending on bacterial adaptation and recovery dynamics.

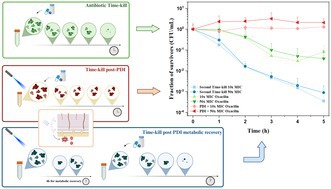

**Abbreviations**
^1^O_2_
: singlet oxygen
AMR: antimicrobial resistance
ATP: adenosine triphosphate
BHI: Brain Heart Infusion
CFU/mL: Colony‐Forming Units per milliliter
CUR: curcumin
MDK: minimum duration of killing
MIC: minimum inhibitory concentration
MRSA: methicillin‐resistant *S. aureus*
PBS: phosphate buffered saline
PDI: photodynamic inactivation
PS: photosensitizer
RNS: reactive nitrogen species
ROS: reactive oxygen species
SOD: superoxide dismutase
WHO: World Health Organization

## INTRODUCTION

Microbial resistance constitutes a major global public health concern, as highlighted by the World Health Organization (WHO).^1^ The WHO reports that infections caused by resistant bacteria significantly contribute to the growing threat of antimicrobial resistance (AMR), which could result in up to 10 million deaths annually by 2050 if effective measures are not implemented.^2^, ^3^ Bacterial resistance has been documented since the discovery of antibiotics, involving mechanisms such as genetic mutations, horizontal gene transfer, and modifications of antibiotic targets.^3^, ^4^, ^5^ Beyond resistance, phenomena such as tolerance and persistence also play critical roles in antibiotic treatment failure.

Tolerance is a population‐wide phenomenon that prolongs bacterial survival under lethal antibiotic exposure in the absence of classical resistance mechanisms. Studies have shown that this extended survival period offers a critical window for the evolution of resistance.^6^, ^7^ Moreover, tolerance can enhance survival under protection against multiple classes of antibiotics, as it is not mediated by specific resistance determinants targeting individual antibiotic types.

Persistence is a subset of tolerance, in which only a small fraction of the population exhibits the tolerant phenotype. It is characterized by biphasic killing curves: an initial rapid death of susceptible bacteria followed by a slowed death rate, allowing persisters to survive prolonged antibiotic exposure, even in the presence of high antibiotic concentrations. In addition, bacterial populations derived from persister survivors exhibit the same biphasic survival curve when treated again, indicating that persistence is not a heritable trait, but rather a transient, phenotypic adaptation.^4^, ^8^

A key parameter in treating persistent bacteria is the minimum duration of killing (MDK), which denotes the time required to eradicate a significant portion of a microbial population.^9^ In the initial phase, the MDK_99_, defined as the time needed to kill 99% of the population, is similar to that of sensitive bacteria, indicating that a substantial portion of the population is eliminated quickly. However, the death rate drastically slows in the second phase, reflecting the presence of a persistent subpopulation. At this stage, the MDK 99.99% of the population (MDK_99.99_) is significantly extended relative to populations for sensitive populations. This shift serves as a persistent phenotype marker, indicating that these cells can survive for prolonged periods even under high antibiotic concentrations. When using concentrations far above the minimum inhibitory concentration (MIC), it is possible to evaluate the weak dependence of persister killing rates on antibiotic levels, attributed to a metabolic dormancy state. In this state, essential biological processes such as cell division and protein synthesis are reduced or halted, thereby reducing the efficacy of antibiotics that target actively growing cells, which typically rely on metabolically active cells.^4^

Persistent bacterial populations contribute to recurrent or chronic infections, as seen in *S. aureus*–related diseases. *S. aureus*, a Gram‐positive bacterium, is one of the most prominent opportunistic human pathogens, capable of adapting to adverse conditions and causing severe infections such as endocarditis, pneumonia, meningitis, sepsis, and osteomyelitis.^10^ These infections can be acute, recurrent, or persistent. The evolution of pathogens in cases of antimicrobial resistance and persistent infections occurs within the host environment, where additional selective pressures, such as immune responses and antibiotic exposure, can contribute to both drug resistance development and immune system evasion.^10^, ^11^

One adaptive mechanism in *S. aureus* involves macrophage‐induced antibiotic tolerance, associated with the oxidative and nitrosative stress generated during phagocytosis (ROS and RNS). Upon phagocytosis, macrophages generate various reactive oxygen species (ROS) and reactive nitrogen species (RNS), such as superoxide, hydrogen peroxide, nitric oxide, and peroxynitrite, which can damage cellular structures and promote bacterial killing. However, *S. aureus* has a robust antioxidant defense system, including superoxide dismutases (SodA, SodM), catalase, and antioxidant molecules such as staphyloxanthin, enabling survival within macrophages. Notably, bacteria that withstand this hostile environment develop near‐complete tolerance to bactericidal antibiotics.^10^, ^12^

Photodynamic inactivation (PDI) represents a promising strategy for microbial eradication through the generation of ROS, which induce oxidative stress within bacterial cells.^13^, ^14^ PDI is based on the interaction between light and photosensitizing agents, resulting in the formation of singlet oxygen and other ROS that compromise essential cellular structures and functions.^15^

Upon irradiation with light of a specific wavelength, the PS transitions to an excited state and can engage in two types of photochemical reactions: Type I and Type II.^16^ In the Type I mechanism, electron transfer occurs between the excited triplet state of the PS and surrounding biomolecules, forming radical species that subsequently react with molecular oxygen to produce ROS. In Type II reactions, energy is directly transferred from the excited PS to molecular oxygen, yielding singlet oxygen (^1^O_2_), the primary cytotoxic species responsible for photodynamic damage.^15^ These processes culminate in oxidative modifications of proteins, lipids, and nucleic acids, ultimately leading to microbial cell death.^17^

Bacteria possess antioxidant defense systems—including superoxide dismutase (SOD), catalase, and peroxidases—to counteract ROS. However, PDI overwhelms these mechanisms via excessive ROS generation, resulting in irreversible cellular damage.^18^ Notably, singlet oxygen generated through Type II reactions is particularly deleterious, as conventional antioxidant defenses are ineffective against this species.^19^ Photosensitizers such as porphyrins, phthalocyanines, and phenothiazines (e.g., methylene blue) exhibit high quantum yields for singlet oxygen production, whereas photosensitizers like curcumin predominantly generate free radicals and hydrogen peroxide.^20^, ^21^

Curcumin, a polyphenolic compound derived from turmeric (*Curcuma longa*), has low toxicity and is safe for food, medical, and environmental applications. It exhibits a broad absorption spectrum in the visible range (340–535 nm), making it compatible with widely available and cost‐effective blue light sources. It demonstrates broad‐spectrum antibacterial activity against both Gram‐positive and Gram‐negative bacteria, including *S. aureus*, *Enterococcus faecalis*, *Staphylococcus intermedius*, *Bacillus subtilis*, *Escherichia coli*, and *Salmonella typhimurium*.^22^ The combination of PDI and antibiotic administration has shown synergistic effects, enhancing bacterial eradication beyond conventional antibiotic therapy, and offering an alternative to the treatment of resistant bacteria.^23^, ^24^ On photoactivation, curcumin generates ROS via both type I (free radical‐mediated) and type II (singlet oxygen‐mediated) pathways. However, it has low singlet oxygen (^1^O_2_) yield, generating predominantly type I ROS, such as free radicals (superoxide $O_{2}^{\cdot -}$ and hydroxyl radical OH·) and hydrogen peroxide (H_2_O_2_), known to induce a tolerance state in *S. aureus* by stimulating the bacteria to enter dormancy or reduce their metabolic activities, enhancing bacterial cell survival to antibiotic treatment.^10^, ^20^, ^21^ As such, it is of interest to evaluate the effect that subinhibitory doses of curcumin‐mediated PDI may promote in combination with antibiotic treatment, as, in this context, PDI may serve both as a promising tool to combat persistent bacterial populations or hinder antimicrobial efficacy. Therefore, this study aimed to analyze the persistence behavior of an *S. aureus* strain and evaluate the effects of PDI treatment with curcumin on bacterial survival and killing kinetics.

## MATERIALS AND METHODS

### Bacterial strains

*Staphylococcus aureus* (ATCC 25923) and a clinical isolate of methicillin‐resistant *S. aureus* (MRSA) were used. Bacterial strains were stored at −80°C in brain heart infusion (BHI) broth supplemented with 20% glycerol. Inoculum was prepared by thawing bacterial stocks, followed by incubation in BHI at 37°C with agitation (0.125×g) in a shaker incubator (Quimis®). Cells were subsequently centrifuged at 3000×g, washed twice with phosphate buffered saline (PBS), and resuspended in PBS. The optical density at 600 nm (OD600) was measured using a spectrophotometer (Cary UV‐Vis50, Varian) to adjust the bacterial concentrations to approximately 10^7^–10^8^ colony‐forming units per milliliter (CFU/mL).

### Bacterial persistence assays

#### MIC

The MIC was determined according to Clinical and Laboratory Standards Institute (CLSI)^25^ guidelines using Oxacillin. Serial dilutions of Oxacillin were prepared in 96‐well plates using Mueller–Hinton broth. Bacterial suspensions were adjusted to a final concentration of 10^6^ CFU/mL, and positive (growth control) and negative (medium‐only) controls were included. Plates were incubated at 37°C for 24 h. Following incubation, resazurin solution (0.002%) was added to each well and incubated for 4 h at 37°C to determine MIC, defined as the lowest antibiotic concentration inhibiting visible bacterial growth.

#### Time‐kill assay

Overnight cultures were diluted 1:200 in Mueller–Hinton broth and incubated at 37°C with agitation (0.125×g) for 3 h to reach mid‐exponential phase. Samples were adjusted to 10^8^ CFU/mL and treated with oxacillin at 10× and 50× MIC. At predefined time points, bacterial cultures were centrifuged, washed, serially diluted (10^−1^ to 10^−6^), and plated on Mueller–Hinton agar for CFU/mL determination. The stationary phase of the time–kill curve was analyzed to identify persistent cells, and survivors were collected for hereditary testing.

#### Hereditary testing

Overnight cultures were diluted 1:200 in Mueller–Hinton broth and incubated at 37°C with agitation (0.125×g) for 3 h. Cultures were then exposed to Oxacillin and incubated for 24 h to induce partial population lysis. Surviving cells were centrifuged (3000×g, 5 min), resuspended in fresh BHI broth, and incubated at 37°C with agitation for 16–24 h. This new population of persistent cells was retested for antibiotic survival. This procedure was repeated three times.

#### Growth phase dependence of persistence formation

Overnight cultures were diluted 1:200 in BHI and incubated at 37°C with agitation (0.35×g). At different growth phases (lag, log, and stationary), cultures were treated with oxacillin to induce persistence and incubated at 37°C with agitation for 21 h. Samples were collected pre‐ and post‐antibiotic treatment, centrifuged, serially diluted, and plated for CFU/mL determination. The frequency of persister cells was compared across growth phases, including under PDI conditions.

### PDI

A stock solution of curcumin (5 mM) was prepared in ethanol and diluted in distilled water to obtain final concentrations of 2.5, 2.0, 1.5, and 1 μM. Experimental groups included a general control (bacteria + PBS), a dark control (bacteria + curcumin no light), a light control (bacteria + light no curcumin), and a PDI‐treated group (bacteria + curcumin + light). In the PDI and dark control groups, 500 μL of bacterial suspension was mixed with 500 μL of curcumin solution in a 24‐well plate and incubated in the dark at 37°C for 15 min. After incubation, the light control and PDI groups were irradiated with 5.0 or 2.5 J/cm^2^ using a 450 nm LED‐based device (Biotable®) with a power output of 40 mW.

Light dose was calculated using Equation (1):

(1) D=I.t

*D*, dose (fluence); *I*, light intensity (mW/cm^2^); *t*, irradiation time (*s*).

Samples were serially diluted and plated to determine CFU/mL post‐treatment. Surviving bacteria at the selected dose and irradiation conditions were centrifuged and resuspended in Mueller–Hinton medium before being transferred to Falcon tubes containing 10× and 50× MIC of oxacillin. Samples were collected, diluted, and plated hourly to assess CFU/mL, following the established time‐kill methodology. Similarly, a second time‐kill assay was performed after metabolic recovery of the PDI‐surviving population. The microorganism was kept under agitation and at optimal growth temperature for 4 h to allow metabolic restoration and subsequently following the same time‐kill assay methodology described above.

### Data analysis

Data analysis was guided by the study's specific objectives, focusing on reducing bacterial survival times (MDK) using oxidative photodynamic methods. The experiments were performed in independent triplicates (*N* = 9) for each group studied. Results were evaluated based on microbial inhibitory effects observed under PDI treatment and analyzed by ANOVA associated with post hoc Tukey test; the error bar was determined by standard deviation. A *p*‐value < 0.05 was considered statistically significant, and Origin Version 2020 was used for plotting the graphics.

## RESULTS

### Bacterial persistence characterization

#### MIC

The MIC of oxacillin for the *S. aureus* strain analyzed was determined to be 0.125 μg/mL, indicating susceptibility, according to Clinical and Laboratory Standards Institute (CLSI)^25^ guidelines. The MIC of oxacillin for the MRSA strain analyzed was determined to be 64 μg/mL, classifying it as resistant according to CLSI guidelines. Based on the found MIC, concentrations of 10× and 50× MIC were selected for the time‐kill assays.

#### Time–kill assay

**FIGURE 1:**
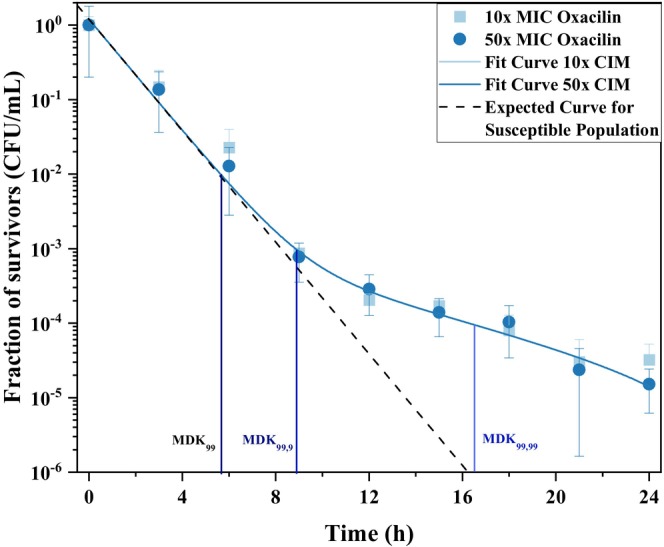
Time–kill curve of *Staphylococcus aureus* using 10× and 50× MIC of oxacillin. The fitted curves for the data points collected over 24 h at 10× and 50× MIC exhibit the typical biphasic behavior of populations with persistent phenotypes. The dashed curve represents the expected behavior for sensitive bacterial populations. Created by the authors.

Time‐kill assays were conducted to determine whether the studied bacterial population exhibited persistence characteristics (Figure 1). For the *S. aureus* strain, the observed killing curves displayed a biphasic pattern, which is indicative of a persistent subpopulation containing persistent phenotypes. The initial phase showed rapid elimination of susceptible cells, followed by a second phase where the death rate slowed considerably, allowing a subpopulation of bacteria to survive prolonged antibiotic exposure.

The biphasic nature of the killing curves suggests the presence of a metabolically dormant subpopulation capable of enduring prolonged antibiotic exposure. The same pattern was observed at both 10× and 50× MIC, which supports the idea that persistence is independent of antibiotic concentration beyond a certain threshold. This survival mechanism is linked to metabolic dormancy, where essential cellular processes, such as division and protein synthesis, are downregulated, reducing the efficacy of antibiotics that target metabolically active cells.^4^

The analysis of time–kill data also showed that the minimum duration for killing 99% of the population (MDK_99_) was comparable to that of fully susceptible strains, indicating that a significant portion of the population was eliminated rapidly. However, in the persistent subpopulation, the MDK_99.99_ was significantly extended, demonstrating that these cells can survive for extended periods even in the presence of elevated antibiotic concentrations. This temporal shift in bacterial survival further is consistent with the persistence phenotype.

**FIGURE 2:**
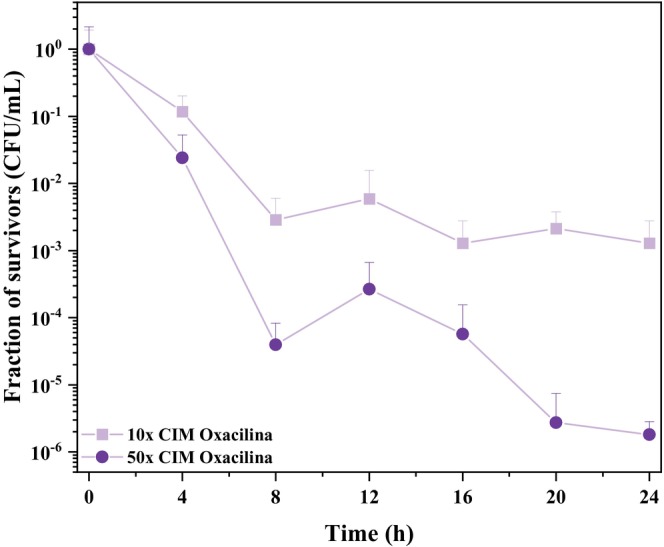
Time–kill curve of *Staphylococcus aureus*‐MRSA using 10× and 50× MIC of oxacillin. The fitted curves for data points collected over 24 h at 10× and 50× MIC exhibit biphasic behavior associated with populations containing persistent phenotypes. Created by the authors.

The MRSA strain exhibited a biphasic survival curve, similar to the susceptible strain (Figure 2). However, differences were observed between the 10× and 50× MIC conditions, suggesting that the survival of MRSA may be influenced by heteroresistance rather than persistence. The distinct killing dynamics observed at different antibiotic concentrations indicate that a subpopulation might be adapting to high oxacillin concentrations rather than entering a metabolically dormant state.

#### Heritability of persistence

**FIGURE 3:**
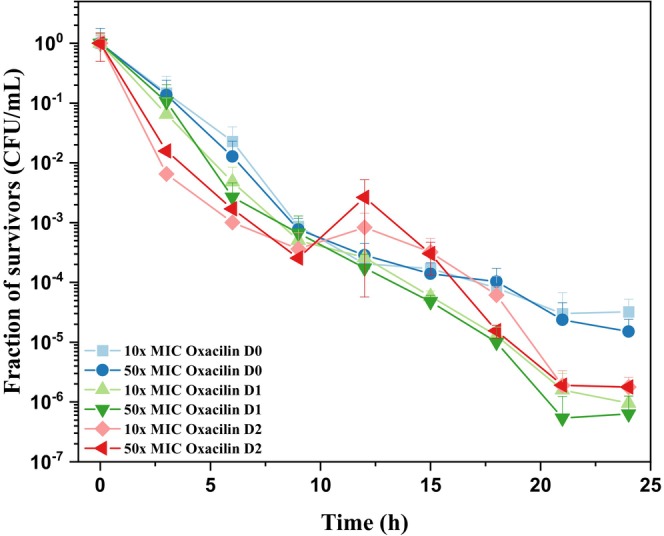
Time–kill curve of *Staphylococcus aureus* using 10× and 50× MIC of oxacillin. D0: Day 0, sensitive population; D1: Day 1, population derived from persister survivors of D0; and D2: Day 2, population derived from persister survivors of D1. Created by the authors.

To assess whether persistence is an inherited trait, surviving bacterial populations from the time–kill assays were isolated and re‐exposed to Oxacillin for three consecutive days (Figure 3). The results showed that the derived populations retained the biphasic killing profile without an increase in the proportion of persisters. These findings indicate that, as expected for the persistence phenotype, the population maintains a reversible phenotypic state in which bacterial cells do not pass on the dormant state to their progeny. Together with the previous results, this confirms that the analyzed population maintains persistence characteristics. This conclusion is further supported by the absence of MIC alterations compared to susceptible bacteria, excluding the possibility of heteroresistance.

#### Growth phase dependence

**FIGURE 4:**
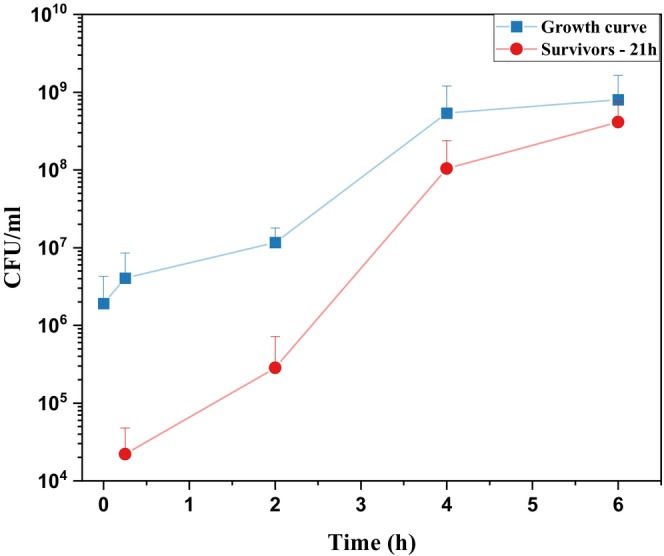
Growth curve of *Staphylococcus aureus* over 6 h and persister survivors after treatment with 50× MIC of oxacillin for 21 h. Created by the authors.

To investigate whether persistence formation depends on bacterial growth phase, cultures were harvested at different growth phases (lag, early log, late log, and stationary) and exposed to antibiotic treatment (Figure 4). The analysis of bacterial killing kinetics showed a marked increase in the fraction of persister cells at the end of the exponential phase and in the stationary phase. This suggests that rapid growth conditions may favor the formation of these subpopulations, with the greatest abundance of persisters observed in the stationary phase. These findings support the hypothesis that factors such as nutrient limitation and high population density contribute to persister cell emergence, as previously described in other studies.^26^ These results indicate that persister formation is tightly associated with the metabolic state of the bacterial population and is influenced by the stage of the growth cycle.

### PDI optimization

**FIGURE 5:**
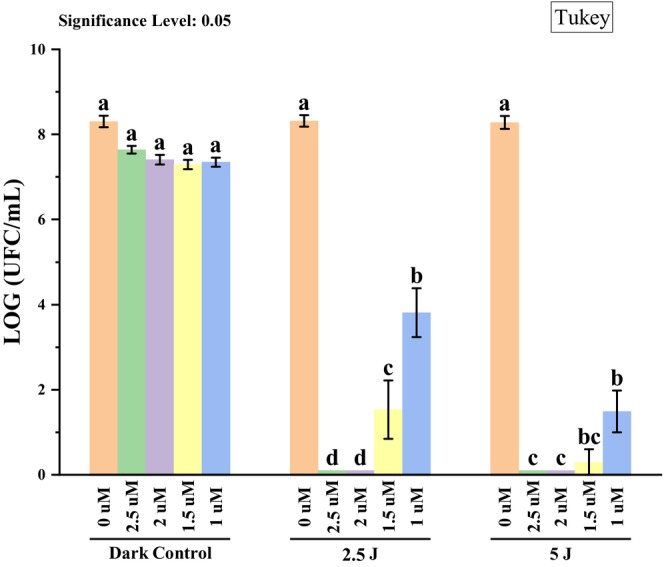
PDI graph showing *Staphylococcus aureus* survival in dark controls with curcumin concentrations ranging from 2.5 to 1 μM, light dose controls at 2.5 and 5 J/cm^2^, and PDI with fixed light doses for different curcumin concentrations. Bacterial load was undetectable for concentrations of 2 and 2.5 μM at both light doses. Different letters indicate statistically significant differences (*p* < 0.05). Created by the authors.

The effects of different curcumin concentrations (1.0–2.5 μM) and light doses (2.5 and 5.0 J/cm^2^) on bacterial survival were analyzed (Figure 5). Control groups (dark and light‐only conditions) showed no significant bacterial reduction, demonstrating that bacterial inactivation requires both curcumin and light exposure. A dose‐dependent response in bacterial load was observed, with a 4.5‐log reduction at 1 μM curcumin and 2.5 J/cm^2^. At 2 and 2.5 μM curcumin, bacterial counts were below the detection threshold for both light doses, indicating complete eradication. These results confirm the high efficacy of PDI when curcumin concentration exceeds 2 μM. After these tests, the treatment group that resulted in the lowest reduction in bacterial viability, 1 μM curcumin and 2.5 J/cm^2^, was selected for additional time–kill assays to assess bacterial killing kinetics following PDI.

### Effect of PDI on persistence

#### Time–kill assay

**FIGURE 6:**
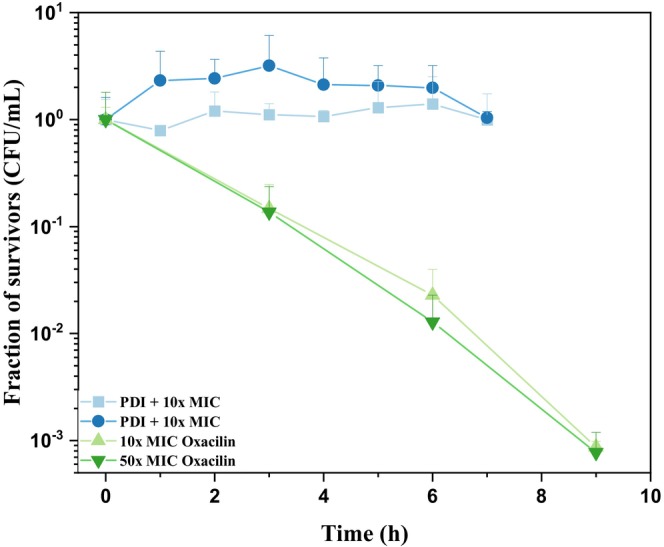
Time–kill curve of *Staphylococcus aureus* using 10× and 50× MIC of oxacillin after PDI application (1 μM and 2.5 J/cm^2^). Created by the authors.

To evaluate whether PDI affected the formation of persister cells, time–kill assays were conducted on bacterial populations surviving PDI treatment. Figure 6 presents a comparison between the initial population and the post‐treatment population, revealing an altered survival profile, suggesting a behavior associated with tolerance or resistance following PDI. To determine whether this change indicated the presence of resistant bacteria, a new MIC test was performed. The MIC values remained unchanged compared to the original susceptible population, excluding the possibility of acquired resistance. A plausible hypothesis is that this behavioral change may be associated with the role of ROS generated by curcumin during PDI. Curcumin has a low quantum yield for singlet oxygen (^1^O_2_) and predominantly produces ROS^20^, ^21^ that have been previously linked to the induction of tolerance in *S. aureus*.^10^

**FIGURE 7:**
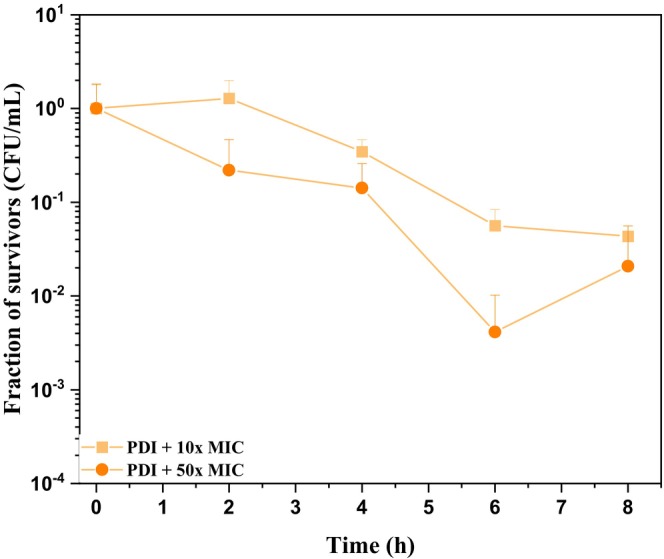
Time–kill curve of *Staphylococcus aureus*‐MRSA using 10× and 50× MIC of oxacillin after PDI application (1 μM and 2.5 J/cm^2^). Created by the authors.

Post‐PDI time–kill assays on the methicillin‐resistant strain (Figure 7) showed no observable increase in tolerance, suggesting that MRSA's resistance mechanisms provide adequate protection against oxidative stress. This indicates that, unlike in susceptible strains, PDI may be ineffective in inducing dormancy‐associated tolerance in MRSA. Further studies are needed to explore the interactions between PDI and resistance mechanisms in resistant bacterial populations.

#### Growth phase dependence

**FIGURE 8:**
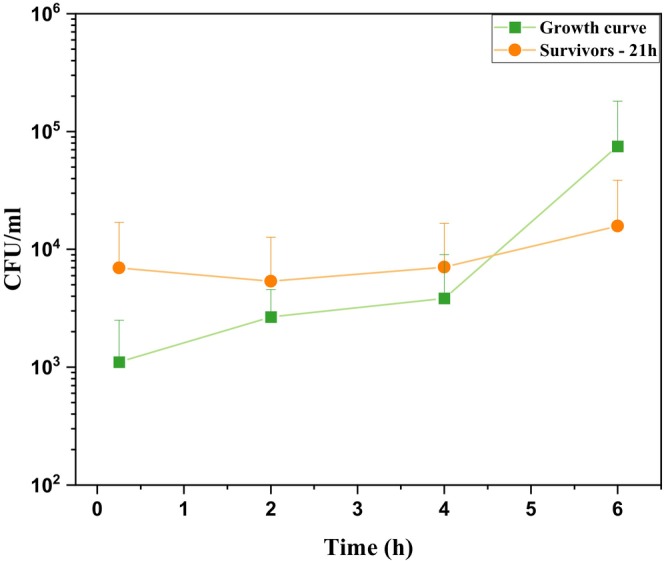
Growth curve of *Staphylococcus aureus* after PDI treatment over 6 h and persister survivors after treatment with 50× MIC of oxacillin for 21 h. Created by the authors.

In addition, an additional experiment was performed to analyze growth dynamics and persister formation following PDI treatment. The objective was to evaluate how initial PDI exposure influenced bacterial population behavior over time, focusing on recovery dynamics and persister frequency. Figure 8 presents the growth curve of bacterial populations treated with PDI compared to surviving bacteria following 21 h of antibiotic treatment. During the first 4 h after PDI treatment, the population exhibited a significant growth delay, indicating that PDI‐induced oxidative stress transiently impaired cell viability. This observation supports the hypothesis that treatment‐induced stress may trigger metabolic dormancy and antibiotic tolerance in bacterial cells. However, after 6 h, a marked increase in bacterial growth was observed, indicating transition out of the dormant state and recovery of metabolic activity. Persister cell formation remained stable up to 4 h post‐PDI treatment, followed by an abrupt decrease in the proportion of survivors at the 6‐h mark.

#### Second time–kill assay

**FIGURE 9:**
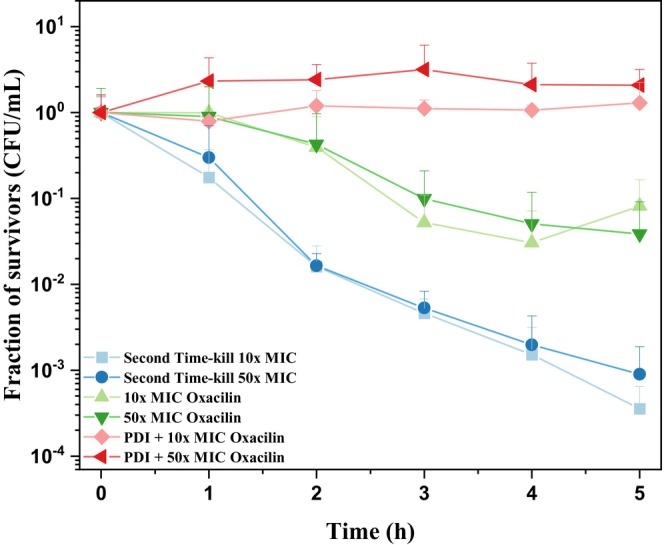
Time–kill curve of *Staphylococcus aureus* using 10× and 50× MIC of oxacillin after recovery of metabolic activity on bacterial populations surviving PDI application (1 μM and 2.5 J/cm^2^). Created by the authors.

Following the assessment of recovery dynamics and persister frequency in the growth dynamics assay, a second time–kill experiment was performed to evaluate the bacterial killing curve after recovery of metabolic activity in populations that had survived PDI treatment. Figure 9 presents a comparison between the killing curves obtained under three conditions: after metabolic recovery of the PDI‐treated population, immediately after PDI exposure, and in the standard time–kill assay without prior PDI. The post‐recovery assay revealed a higher rate of cell death compared with both reference curves, indicating an enhanced combinatory effect of PDI and antibiotic treatment when antibiotics are administered after metabolic restoration, suggesting that residual damage caused by photodynamic treatment can potentiate antibiotic activity once the transient adverse effects on bacterial metabolism have been resolved.

## DISCUSSION

In this study, after determining bacterial susceptibility through the MIC of oxacillin, the analysis of bacterial killing curves revealed a biphasic pattern characteristic of bacterial populations with persistent phenotypes. This pattern highlights the presence of a subpopulation capable of surviving high antibiotic concentrations associated with metabolic dormancy. The consistency of persister cell behavior at both 10× and 50× MIC reinforces the low dependency characteristic persistence of persistence on antibiotic concentration. Unlike resistant cells, which survive through genetic adaptations that enhance their ability to counteract antibiotics, persister cells survive due to their transient, phenotypic state of dormancy. This distinction underscores the importance of alternative therapeutic strategies, such as PDI, which do not depend exclusively on metabolic activity for bacterial inactivation. Additionally, PDI experiments using different curcumin concentrations confirmed its dose‐dependent effectiveness in reducing bacterial load. However, time‐kill assays performed on bacterial populations surviving PDI indicated a potential induction of tolerance in *S. aureus*.

A relevant point of discussion is the hypothesis that this phenotypic alteration may be associated with the role of ROS generated by curcumin during PDI, further supported by the similarity in MIC values before and after PDI. Curcumin has an intrinsically low quantum yield for singlet oxygen (^1^O_2_), with values of 0.01 in ethanol solution, compared to values between 0.5 and 0.8 for photosensitizers such as porphyrins, phthalocyanines, and chlorin derivatives. This means that although curcumin absorbs photons in the visible range, only a small proportion of that energy is converted into singlet oxygen. Thus, curcumin results in lower singlet oxygen generation (^1^O_2_) compared to other photosensitizers used in PDI, generating predominantly ROS, such as free radicals (superoxide $O_{2}^{\cdot -}$ and hydroxyl radical OH·) and hydrogen peroxide (H_2_O_2_).^20^, ^21^

According to Rowe et al.,^10^ ROS can induce a tolerance state in *S. aureus* by stimulating the bacteria to enter dormancy or reduce their metabolic activities, enhancing bacterial cell survival. This effect is attributed to the inactivation of crucial metabolic pathways via oxidative stress generated by ROS, reducing ATP production and placing bacteria in a low‐activity state that may make them temporarily tolerant. Therefore, ROS production by curcumin may have contributed to the induction of a tolerance state in *S. aureus*, where cells surviving PDI treatment entered a dormancy or low metabolic activity state. This hypothesis is further supported by the subsequent time‐kill curve performed after restoration of the population's metabolic activity, which showed an increased rate of cell death compared to both the curve obtained immediately after PDI and the standard time‐kill assay without prior PDI exposure. These findings indicate that the combined use of PDI and antibiotic therapy can be either highly effective or potentially detrimental, depending on the timing and strategy employed for antibiotic administration. A potential test that additionally validates this hypothesis is the application of excess glucose to the medium, which, as performed in Rowe et al.'s^10^ experiment, would increase ATP generation through substrate‐level phosphorylation, consequently restoring antibiotic susceptibility.

During the growth phase dependence assay, the frequency of persister cells remained constant up to 4 h post‐PDI treatment, followed by a sharp decline in survivor proportion at the 6‐h mark. These results suggest that while PDI significantly reduces the initial bacterial load, it may create conditions that promote the selection or induction of persister cells immediately after treatment. Enhanced efficacy of post‐treatment antibiotic administration was observed when administered after bacterial metabolic recovery, preventing the development of tolerance within the population. Future studies should focus on bacterial killing kinetics by introducing antibiotics at different time intervals following PDI treatment.

Resistant *S. aureus* strains exhibited differences in time‐kill curves at 10× and 50× MIC and a gradual decrease in bacterial load in post‐PDI treatment assays. These results may indicate a limitation in tolerance or persistence induction in resistant bacteria. The time‐kill assay results suggest that the observed behavior may not be due to the formation of persistent phenotypes but rather to a heterogeneous resistance adaptation within the population favoring the survival of a subpopulation of cells. This raises the question of whether resistant bacteria might be less prone to persistence formation than susceptible bacteria, as persistence represents a distinct survival mechanism. The distinct behavior observed at 10× MIC may be due to resistant cells adapting to the antibiotic concentration, with a subsequent MIC analysis potentially elucidating an increase in resistance levels. This result suggests that studying killing curves and persistence formation in resistant bacteria may require antibiotic concentrations much higher than those used for susceptible bacteria, such as 100× MIC. To determine whether resistant bacteria are indeed less prone to persistence formation, time‐kill and heritability assays with antibiotics of differing mechanisms of action from those to which the bacteria are resistant would be of interest. The killing curves observed after PDI treatment did not support tolerance induction, possibly due to enhanced adaptation to oxidative stress conditions via bacterial resistance mechanisms or a general limitation in tolerance induction in resistant bacteria, as discussed earlier. As previously mentioned, future research in this area could involve evaluating antibiotics targeting different cellular pathways and initiating time–kill assays at various time points post‐PDI treatment.

An additional point of interest is the metabolic reprogramming in MRSA in response to β‐lactam antibiotic exposure, particularly an increase in TCA cycle activity to optimize energy production. The study by Keaton et al.^27^ demonstrates that MRSA undergoes significant metabolic adaptation when exposed to β‐lactams, redirecting central carbon flux toward the TCA cycle to ensure sufficient energy production for survival. β‐Lactam exposure triggers a complex metabolic reorganization in MRSA, primarily characterized by increased TCA cycle activity to optimize energy production, along with adjustments in other central pathways such as glycolysis, fermentative pathways, carbohydrate metabolism, amino acid metabolism, and fatty acid metabolism, reflecting the bacterium's adaptation to survive under antibiotic selective pressure.^27^, ^28^, ^29^ This metabolic adaptation may be a factor influencing the difference in MRSA behavior in killing curves and after PDI treatment compared to the behavior observed in susceptible *S. aureus*. This adaptation could affect tolerance or persistence behavior in MRSA when treated with β‐lactam antibiotics, reinforcing the need for persistence studies in MRSA using antibiotics to which it is not resistant.

## Data Availability

The data that support the findings of this study are openly available in zenodo at https://doi.org/10.5281/zenodo.16753477.
